# Cancer incidence in immunocompromised patients: a single-center cohort study

**DOI:** 10.1186/s12885-022-10497-4

**Published:** 2023-01-09

**Authors:** Sabrina Ilham, Connor Willis, Kibum Kim, Karen C. Chung, Brenda M. Wood, Malinda S. Tan, Chia Jie Tan, Danielle T. Nguyen, Diana I. Brixner, David D. Stenehjem

**Affiliations:** 1 Department of Pharmacotherapy, College of Pharmacy, L. S. Skaggs Pharmacy Institute, University of Utah, 30 2000 E, Salt Lake City, UT 84112 USA; 2 Department of Pharmacy Systems, Outcomes and Policy, University of Illinois, Chicago, IL USA; 3 GRAIL, LLC, a subsidiary of Illumina, Inc., currently held separate from Illumina Inc, Menlo Park, CA USA; 4 Department of Pharmacy Practice and Pharmaceutical Sciences, College of Pharmacy, University of Minnesota, Duluth, MN USA

**Keywords:** Cancer incidence, Immunocompromised, Transplant, Primary/secondary immunodeficiency, TNF-i

## Abstract

**Background:**

Diminished immune defense plays an important role in cancer development. Cancer risk in immunocompromised patients may differ. Identifying individuals with elevated cancer risk can inform strategies for routine cancer screening. This study aimed to understand and compare cancer incidence and risk in three patient groups: recipients of solid organ transplant (SOT) or hematopoietic stem cell transplant (HSCT); diagnosis of primary or secondary immunodeficiency disorder (PID/SID); and recipients of tumor necrosis factor inhibitor (TNF-i) therapy.

**Methods:**

This retrospective cohort study used the University of Utah Health System database and Huntsman Cancer Institute tumor registry. Patients aged ≥18 years with SOT/HSCT, PID/SID or ≥ 3 months of TNF-i therapy were included. The date of transplant, diagnosis of PID/SID, or 1st TNF-i medication order date was defined as the index date. We calculated cumulative cancer incidence by Kaplan-Meier method. A Cox-proportional hazard regression model with a stepwise variable selection process was used to identify independent risk factors associated with the time to onset of a new primary cancer.

**Results:**

In total, 13,887 patients were included which comprised of 2982 (21%) SOT/HSCT, 7542 (54%) PID/SID and 3363 (24%) patients receiving TNF-i. The mean (SD) age ranged from 46.8 (15) years - 50.4 (18.2) years. The proportion of white patients ranged from 72.3–84.8%. The estimated cumulative cancer incidence was 11.5% in the SOT/HSCT cohort, 14.3% in the PID/SID cohort, and 8.8% in the TNF-i cohort. The multivariable model adjusted for age, benign in-situ disease, Charlson Comorbidity Index, hypertension/cardiovascular disease/end stage renal disease, gender, race/ethnicity, and renal cyst as significant risk factors. The adjusted hazard ratios for cancer development in SOT/HSCT and PID/SID cohorts compared to the TNF-i cohort over the full follow-up period were 1.57 (95% CI: 1.16–2.13) and 2.14 (95% CI: 1.65–2.77), respectively.

**Conclusion:**

A significantly increased risk of cancer was observed in PID/SID patients and SOT/HSCT patients compared to TNF-i patients. Age ≥ 50 years, male gender, and clinical comorbidities were additional factors impacting cancer risk. PID/SID and SOT/HSCT patients may benefit from more intensive cancer screening.

**Supplementary Information:**

The online version contains supplementary material available at 10.1186/s12885-022-10497-4.

## Background

Cancer is currently the second leading cause of death in the United States (US) [1]. Cancer remains the second most costly disease among Americans with a 27% rise in cost within the past 10 years [2–4]. The annual costs of cancer care are estimated to exceed $245 billion dollars by 2030 [5–7]. Delays in cancer detection can lead to decreased functional outcomes, productivity loss, treatment complications, reduced health-related quality of life, and increased healthcare costs [8]. However, cancer screening enables the earlier detection and treatment of cancer which may lead to survival benefits, reduce the complexity of care, and decrease long-term costs in cancer patients [9].

Prior epidemiologic studies suggest that patients with known and severe immune dysregulation following transplant have an increased susceptibility to cancer [10]. A large cohort study linking US transplant registries with 15 population based state and regional cancer registries has found a two-fold increased risk of cancer in transplant patients [11]. Transplant patients have been found to be susceptible to 32 different types of cancer [11], with higher incidence of oncogenic virus-related cancers (e.g., hepatocellular carcinoma, anogenital carcinoma, cervical cancer, GI cancer, head and neck cancer, and nasopharyngeal carcinoma) [12]. Although limited data is available, patients with primary or secondary immunodeficiency disorder (PID/SID) can likewise be at increased risk of cancer [10]. The estimated relative risk of cancer in adults with PID is 1.42 compared with the age-adjusted SEER population [13]. PID is primarily associated with an increased risk of lymphomas, which contributes up to 60% of PID cancer cases [13]. Acquired Immune Deficiency Syndrome (AIDS) is the most common SID, associated with significantly higher rates of cancer especially Kaposi’s sarcoma, non-Hodgkin’s lymphoma, lung, liver, melanoma, or stomach cancer [14, 15]. While immunomodulatory therapy such as tumor necrosis factor inhibitor (TNF-i) may increase the risk of cancer attributable to its mechanism of action, the impact on cancer risk in real-world TNF-i users is still debatable. Current screening recommendations currently do not address the increased cancer risk in immunocompromised patients or the incident cancer types.

Previous studies provide information on cancer risk in specific groups within immunocompromised patients (only solid organ (SOT) or hematopoietic stem cell transplant (HSCT), only PID or SID) [13, 14, 16–18]. However, limited data is available on cancer risk across overall patient groups (combined SOT and HSCT patients or combined PID and SID patients). In addition, little is known regarding the impact of patient characteristics / demographics and pre-existing clinical conditions on cancer risk in patients with dysregulated immune system. The aim of this study was to evaluate and compare cancer risk in three patient groups: recipients of SOT/HSCT, patients with PID/SID and patients treated with TNF-i therapy. As the impact of TNF-i on cancer risk has been found to be non-significant in multiple studies [19–23], we hypothesized that the risk of cancer in SOT/HSCT recipients and PID/SID patients will be higher compared to TNF-i recipients. Data from our study adds insights to the current evidence on the immunosuppression-cancer paradigm and informs strategy for routine cancer screening.

## Methods

This was a retrospective cohort study using electronic health records from the University of Utah Enterprise Data Warehouse (EDW) and data from the Huntsman Cancer Institute Tumor Registry (HCI-TR). The EDW integrates historical and comprehensive health records of more than 1.5 million patients across the University of Utah Healthcare System (hospital and clinics), and the HCI. The HCI-TR registers all primary cancer cases diagnosed and/or treated by the HCI and works closely in cancer tracking activities and statistical reporting procedures with the Utah Cancer Registry, which is a Surveillance, Epidemiology and End Results (SEER) registry.

Overall study cohort included three groups, patients with SOT/HSCT, diagnosis of PID/SID and who received TNF-I therapy between July 1,2000 and February 20, 2018. SOT/HSCT or PID/SID were defined by the Current Procedural Terminology codes and the International Classification of Diseases Ninth and Tenth revision Clinical Modification (ICD-9-CM and ICD-10-CM) codes respectively. The TNF-i subgroup included patients who received at least 3 months of TNF-i therapy. The index date was defined as the date when an individual was first confirmed either as a transplant recipient, diagnosed with PID or SID or initiated TNF-i therapy. The pre-index period was defined from January 1, 2000 to the index date for all patients which allowed for the capture of baseline characteristics and potentially confounding variables [e.g., demographics, prior cancer, comorbidities, Charlson Comorbidity Index (CCI) score]. Patients with age ≥ 18 years at the index date, and with a minimum follow up of 90 days post-index date were included. The cohorts were kept mutually exclusive based on the risk hierarchy as indicated in prior studies [13, 16, 23]: SOT/HSCT patients that met the eligibility criteria for either the PID/SID or TNF-i cohorts were analyzed under SOT/HSCT cohort; PID/SID patients that also received TNF-i therapy, were included in the PID/SID cohort. The TNF-i cohort with presumably lower risk was used as a reference group for comparing risk in other two cohorts.

Following cohort identification, patients were screened through the HCI-TR to identify the first occurrence of a new primary cancer diagnosis (outcome) post-index date. For patients with prior cancer during the pre-index period, a new primary cancer diagnosis was required to meet the definition of the development of cancer. For each cohort, follow-up ended at cancer diagnosis, death, last date of follow-up or end of study period (February 2020) whichever occurred first.

Means with standard deviations (SD) and medians with interquartile ranges (IQR) were reported for continuous variables. Frequencies and percentages were reported for categorical variables. Statistical comparisons were performed using analysis of variance (ANOVA) tests for continuous variables and Chi-square test and Fisher’s Exact test for categorical variables. Differences in the baseline characteristics among three risk groups were considered statistically significant if *P* < 0.05. Number of new cancer cases over observation period were presented in incidence rate per 1000 person-years. To account for the follow-up period in calculating cumulative incidence, we generated Kaplan-Meier product-limit estimates where patients were censored at date of death or last follow-up without the onset of new cancer. We ran univariable Cox regression analysis to identify statistically significant confounders. The potential confounding effects were tested in multivariable Cox regression model. Age (18–49, 50–64, ≥65), BMI (< 18.5, 18.5–24.9, 25–29.9, ≥30) and CCI score (0, 1–2, 3–5, ≥6) were used as categorical variables in the model. Multivariable models were constructed using a stepwise forward selection approach, using *p* < 0.3 as the entry criterion and *p* < 0.2 as the staying criterion. Akaike Information Criteria (AIC) and Bayesian Information Criterion (BIC) were used to compare the prediction errors for nested and non-nested models. Lower values indicated that the model explains data with the consideration of the goodness-of-fit and degrees of freedom. All analyses were performed using the SAS statistical software version 9.4 (Cary, NC: SAS Institute) and STATA statistical software version 16 (College Station, TX: StataCorp LLC.).

## Results

**Fig. 1:**
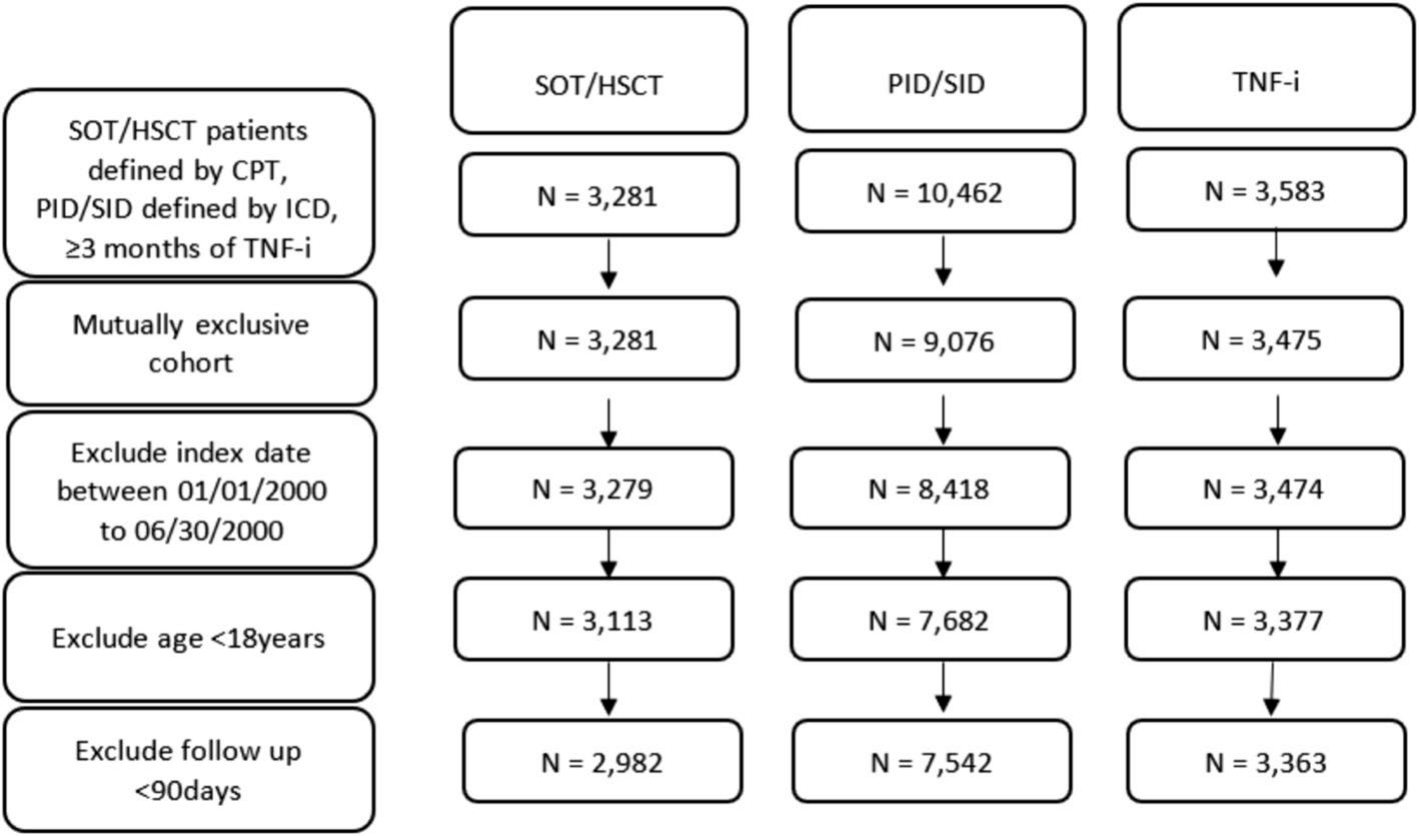
Flow diagram of cohort identification. N = number of patients that remained eligible under each cohort after applying exclusion criteria which are shown sequentially in the left panel

In total, 13,887 patients were included in the analysis: 2982 SOT/HSCT patients, 7542 PID/SID patients, and 3363 patients in the TNF-i cohort (Fig. 1). Out of 2982 transplant patients, 1891 (63.4%) were SOT and 1091 (36.6%) were HSCT patients. The most common SOT type was kidney transplant (60.4%), followed by liver (15.8%) and heart (12.4%). Among HSCT cohort, 710 (65.1%) had autologous and 381 (34.9%) had allogeneic stem cell transplant. The most common type of PID observed (29.4%) was ‘Other Combined Immunodeficiencies’ (ICD-9/10-CM: 277.6/D81.810), followed by ‘Other Specific Disease of Blood and Blood-forming Organs’ (ICD-9/10-CM: 289.89/D89.2) and ‘Unspecified Disorder of Immune Mechanism’ (ICD-9/10-CM: 279.9/D89.9). In the SID cohort, we included patients with HIV (45% of the PID/SID cohort) (ICD-9/10-CM: 042/B20). The median (IQR) duration of exposure to TNF-i was 1.6 years (1.0–3.0). The baseline characteristics of patients by risk cohort are described in Table S1 (Supplemental Material). The proportion of males was higher in the SOT/HSCT and PID/SID cohorts compared to the TNF-i cohort. The average age was higher in the SOT/HSCT (50.3) and PID/SID cohort (50.4) compared to the TNF-i cohort (46.8). Most of the patients in all 3 cohorts were white followed by Hispanic. More than a third of patients in the SOT/HSCT and TNF-i cohort had a BMI ≥30; while a normal BMI (18.5–24.9) was observed in 35.8% of patients in the PID/SID cohort. Prior history of cancer was observed in 38.0% of patients in the SOT/HSCT cohort, followed by 7.6% in the PID/SID cohort and 1.9% in the TNF-i cohort. Most patients (92.3%) in the HSCT cohort had prior history of cancer. The most common baseline cancer(s) in the SOT/HSCT and PID/SID cohort was cancer involving the hematopoietic system while those in the TNF-i cohort were skin, breast, head and neck cancer. Most patients in the SOT/HSCT, PID/SID and TNF-i cohorts had a CCI score of 3–5, 0 and 1–2, respectively. Hypertension (HTN)/ cardiovascular disease (CVD)/ end stage renal disease (ESRD), and diabetes mellitus (DM) were the most common comorbidities observed in all three cohorts. The median (IQR) follow up was 5.5 years (3.0–9.4), 6.2 years (3.7–10.0) and 4.4 years (3.2–6.9) in the SOT/HSCT, PID/SID and TNF-i cohorts, respectively. The baseline characteristics (gender, age, race/ethnicity, BMI), clinical characteristics (history of cancer, 4 categories (≥6, 3–5,1-2,0) of CCI), and most of the comorbidities were significantly different among three cohorts (*p* < 0.01).

**Fig. 2:**
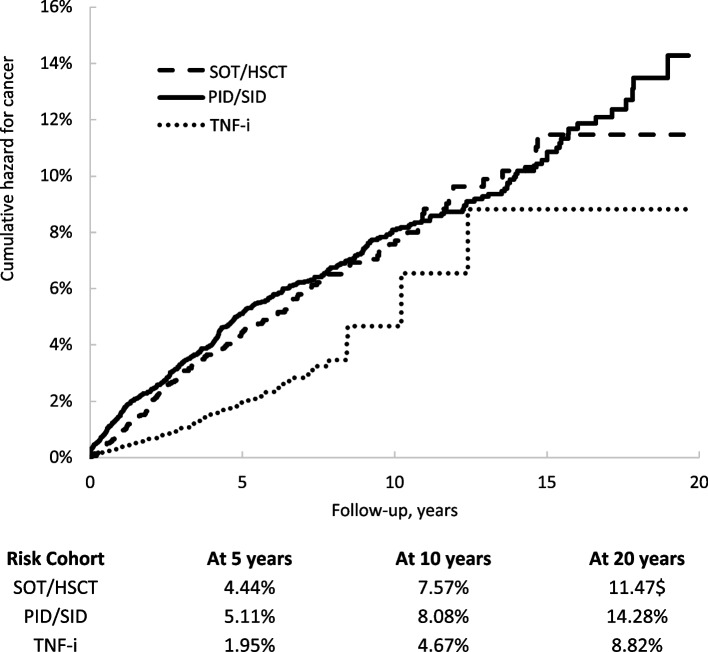
Kaplan–Meier estimates of cumulative hazard of cancer categorized by risk cohorts. Cumulative cancer incidence over 5 years, 10 years and full follow up (20 years) are shown by three risk groups

The estimated cumulative cancer incidence (Kaplan Meier estimates) was 11.5% in the SOT/HSCT cohort, 14.3% in the PID/SID cohort, and 8.8% in the TNF-i cohort (Fig. 2). The crude cancer incidence rate per 1000 person-years for the full follow-up period was 7.5 (95% CI: 6.4–8.7) in the SOT/HSCT, 8.0 (95% CI: 7.3–8.8) in the PID/SID, and 3.4 (95% CI: 2.7–4.3) in the TNF-i cohort. The unadjusted hazard ratios for cancer in the SOT/HSCT and PID/SID cohorts compared to the TNF-i cohort over the full follow-up period were 2.16 (95% CI: 1.63–2.87) and 2.32 (95% CI: 1.80–3.00), respectively.

From the analysis of overall cohort, female patients (compared to male) [HR: 0.81 (95% CI: 0.70, 0.94)], patients of unknown/other race or ethnicity (compared to white) [HR:0.50 (95% CI: 0.32, 0.79)], and with CCI of 0 (compared to CCI 1–2) [HR: 0.74 (95% CI: 0.62, 0.90)] were associated with a lower risk of cancer onset. Patients with BMI 25–29 had 23% higher risk of cancer onset [HR: 1.23 (95% CI: 1.01, 1.48)] compared to the patients with BMI 18.5–24.9. Prior history of a cancer was significantly associated with cancer risk in the overall cohort [HR: 1.38 (95% CI: 1.11, 1.71)]. Age was the only covariate that was consistently associated with cancer risk across all 3 cohorts, with a significantly higher risk among patients ≥50 years old compared to those 18 to 49 years old [HR:3.04 (95% CI: 2.57, 3.59)]. In addition, patients who had benign in-situ disease [HR:2.28 (95% CI: 1.82, 2.85)], DM [HR:1.4 (95% CI: 1.13, 1.73)], HTN/CVD/ESRD [HR:1.6 (95% CI: 1.37, 1.88)], COPD/asthma/pneumonia/bronchitis [HR:1.55 (95% CI:1.21, 1.99)], use of diuretics [HR:2.67 (95% CI:1.27, 5.61)] and renal cyst [HR:2.54 (95% CI:1.51, 3.66)] had a higher risk of cancer compared to those who did not have those comorbidities (Table S2) (Supplemental Material).

**Fig. 3:**
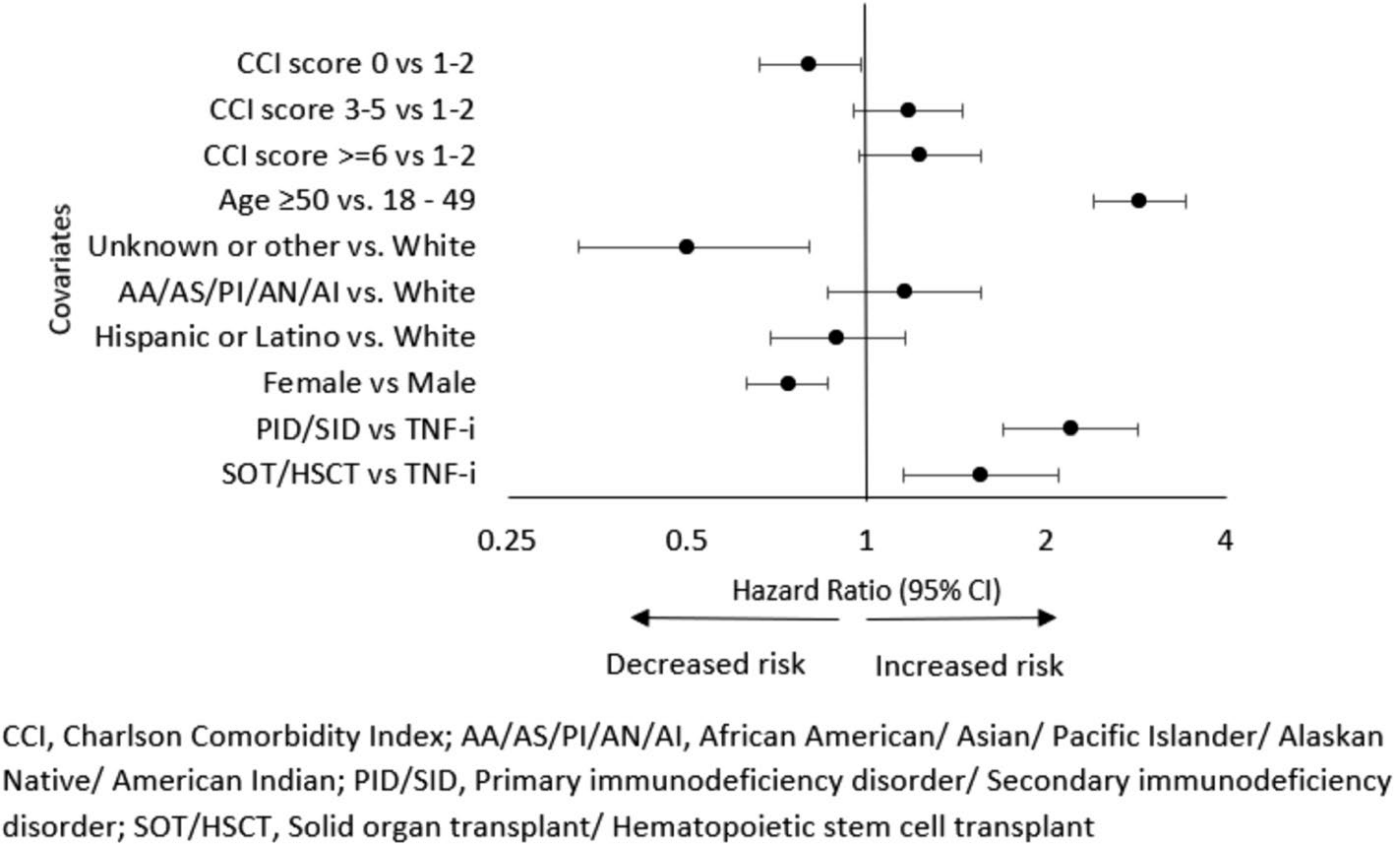
The hazard ratio estimates of potential predictors of cancer among all the enrollees after Cox Multivariable Regression Analysis. Values greater than 1 indicate increased cancer risk and vice versa. The left panel shows the predictor variables and their corresponding comparators

Due to potential multicollinearity between comorbidities (CVD, ESRD, DM, COPD, pneumonia, bronchitis), baseline cancer and conditions used to calculate CCI score (cancer, metastatic cancer, myocardial infarction, congestive heart failure, peripheral vascular disease, renal disorder, DM, pulmonary disease), two multivariable models were developed. In one model, we adjusted for age, race/ethnicity, gender, BMI and CCI score. Controlling for other potential predictors, individuals with SOT/HSCT had 55% [HR:1.55 (95% CI: 1.15, 2.10)] and PID/SID had 121% [HR: 2.21 (95% CI: 1.70, 2.86)] higher risk of cancer compared to recipients of TNF-i therapy. Age was the only independent predictor selected by the model [HR:2.88 (95% CI: 2.41, 3.43)] (Fig. 3).

**Fig. 4:**
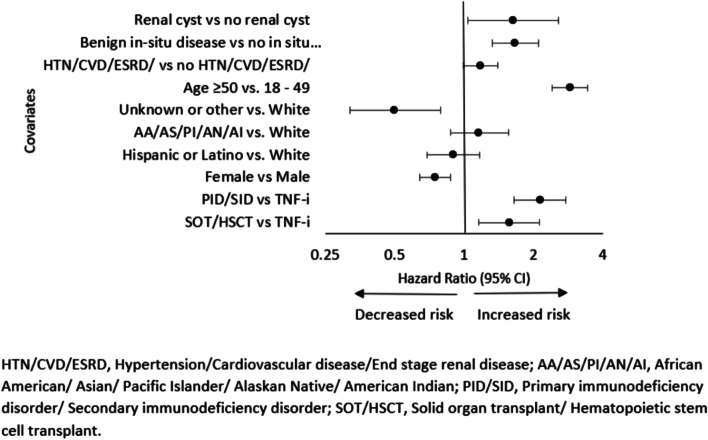
The hazard ratio estimates of potential predictors of cancer among all the enrollees after Cox Multivariable Regression Analysis. Values greater than 1 indicate increased cancer risk and vice versa. The left panel shows the predictor variables and their corresponding comparators

The second model adjusted for age, race/ethnicity, gender, baseline cancer, HTN/CVD/ESRD, DM, COPD/asthma/pneumonia/bronchitis, DM, renal cyst and benign in-situ disease. Controlling for other potential predictors, individuals with SOT/HSCT had 57% [HR:1.57 (95% CI: 1.16, 2.13)] and PID/SID had 114% [HR: 2.14 (95% CI: 1.65, 2.77)] higher risk of cancer compared to recipients of TNF-i therapy. In addition, age ≥ 50 years [HR: 2.89 (95% CI: 2.42, 3.44)], history of benign in-situ disease [HR: 1.67 (95% CI: 1.33, 2.11] and renal cyst [HR: 1.64 (95% CI: 1.04, 2.56] were significant predictors of cancer risk (Fig. 4).

A total of 701 patients developed new primary cancer; 161 in the SOT/HSCT cohort, 471 in the PID/SID cohort and 69 in the TNF-I cohort (Table S3). In the SOT/HSCT cohort, GI cancer (14.3%) was the most common newly diagnosed cancer followed by cancer of hematopoietic system and skin cancer (12.4% each). In the PID/SID cohort, cancer of hematopoietic system was predominant (22.3%) followed by GI (16.8%) and skin cancer (14.2%). In the TNF-i cohort, cancer of reproductive system (17.4%) was the most common followed by breast cancer (15.9%).

## Discussion

In our analysis of real-world data of 13,887 individuals, the risk of cancer was significantly higher in patients with PID/SID or SOT/HSCT relative to patients who received TNF-i therapy while controlling for other predictors of cancer risk. The crude incidence rate of cancer estimated in these two cohorts (PID/SID: 8.01 cases per 1000 person-years; SOT/HSCT: 7.48 per 1000 person-years) was almost twice as high as the age-adjusted cancer incidence in the region, (4.02 cases per 1000 person-years) [24]. The crude cancer incidence in the TNF-i cohort was 3.45 cases per 1000-person years which is close to the age-adjusted incidence in Utah. Similar trends in the escalation of cancer risk among transplant patients have been reported in the literature [11]; however, the increase in cancer risk among patients with PID/SID in our study appeared to be larger than findings from previous studies (RR:1.42 compared to SEER age-adjusted population) [13]. This higher estimate may be a reflection of overall risk from a combined PID/SID cohort compared to only PID patients in the prior study [13]. Another reason of this discrepancy may be heterogeneity in causes of immunodeficiency, which can influence cancer risk. In addition to the specific PID disorders that have shown to increase the risk of cancer (common variable immunodeficiency disorder, severe combined immunodeficiency disorder, Wiskott-Aldrich syndrome, hypogammaglobulinemia) [13, 25], the PID/SID cohort in this study also captured a variety of additional PID disorders (e.g., other common immunodeficiencies, other disorders of blood and blood forming organs, and unspecified disorder of immune mechanism etc).

Baseline demographics and clinical characteristics among three groups can influence cancer risk in three cohorts. To adjust for selection bias, we incorporated the clinically and statistically relevant variables e.g., age, gender, race/ethnicity, body mass index, prior history of cancer and certain comorbidities (e.g., cardiovascular disease, diabetes, benign in-situ- disease etc.) in our regression analysis. In the multivariate model while controlling for age, sex and other confounders, cancer risk in the SOT/HSCT and PID/SID cohort was significantly higher than the TNF-i cohort. Consistent with trends reported in the literature [26], age was found to be associated with cancer risk, with an effect size consistent across cohorts. Therefore, cohorts with a higher proportion of elderly patients (e.g., PID/SID and SOT/HSCT) possibly had a higher incidence of cancer. Similarly, cohorts with a higher proportion of males (SOT/HSCT) might have higher incidence of cancer as both the univariable and multivariable model found a lower risk of cancer in females. In addition, a history of benign or in-situ growth was associated with an increased cancer risk in the overall cohort. The progression of benign growths to malignant cancers is most studied among women with benign breast disease, who have been found to have more than twice the risk of developing subsequent breast cancer compared to their counterparts who do not have benign breast disease [27]. A similar association with higher cancer risk was also observed with a history of renal cysts, which have a risk of developing into renal cell carcinomas [28, 29].

One of the indications of organ transplantation (SOT and most cases of HSCT) is organ failure from cancer. HSCT is a recommended treatment for several types of hematologic cancers, e.g., acute leukemia, aggressive B cell lymphoma, multiple myeloma [30]. That’s why we included patients with prior history of cancer in our cohort to have a representation of real-world transplant patients. As patients with prior history of cancer may develop subsequent cancer of different types, prior history of cancer was included as one of the variables in our regression model. Although prior history of cancer was a statistically significant predictor of cancer in the univariate analysis, the effect was confounded by some other predictors e.g., age, benign disease in the multivariable model. In addition to prior history of cancer, other factors that may be associated with increased cancer risk in transplant especially HSCT patients include, age at HSCT, pre-HSCT exposure to chemotherapy and radiation, infection with oncogenic viruses (Epstein–Barr virus [EBV] and hepatitis B and C viruses), autologous versus allogeneic HSCT etc. [31].

There has been a lack of evidence to suggest an increased risk of cancer among patients who receive TNF-i therapy, both in our study cohort and in the literature [32]. Biologically, the role of TNF-alpha in cancer progression remains unclear and has been linked to both cancer-suppressing and -promoting pathways [33]. Therefore, insights from real-world evidence may shed light on the effect of long-term TNF-i therapy on cancer risk, guiding the clinical use of TNF-i agents especially among individuals who are already predisposed to higher risk of cancer.

The types of incident cancer observed in the SOT/HSCT and PID/SID cohorts (e.g., GI, cancer of hematopoietic system and skin cancer) support the possible association of immunodeficiency and subsequent oncogenic virus-or bacterial infection related cancer. GI cancer can be associated with Epstein Barr virus (EBV) or *Helicobacter pylori* infection [13, 16]. Increased risk of lymphoma and leukemia can be related to EBV driven lymphoproliferation [13]. Three pathogenic human viruses have been linked with skin neoplasms; human papilloma virus, Kaposi’s sarcoma associated herpes virus and human T cell leukemia virus type 1. Viruses can alter keratinocytes by activation of cancer promoting genes and accelerated viral carcinogenesis is mostly observed in immune deficient hosts [34]. Cancer of reproductive system and breast cancer was identified most frequently in the TNF-i cohort along with skin cancer. The most common cancers of the reproductive system such as, cervical and anal cancer may be associated with human papilloma virus [35]. Also, the TNF-i cohort comprised more than 60% of females and females are more susceptible to breast cancer and cancer of the reproductive system. Based on the incident cancer types, a broader cancer screening approach may be warranted in this patient population as over 50% of the cancers in the SOT/HSCT and PID/SID population would not be detected through current cancer screening recommendations (i.e., breast, lung, colorectal, cervical, prostate).

Findings of our research should be considered in the context of the retrospective observational research design, which is subject to misclassification due to the coding, incomplete records, and unobserved confounders. Patient characteristics differed significantly between the cohorts, potentially complicating comparisons between the groups of patients. To address this limitation, covariates that potentially influence the onset of cancer were controlled for in our analysis. The differences in the size of the cohorts may affect the statistical significance of the associations between the covariates, exposure, and outcomes. In addition, we could not adjust for some of the highly reported risk factors, (e.g, smoking, alcohol history, family history) as the database did not provide enough information to capture these variables appropriately. Using data specific to Utah population may limit the generalizability of our result.

## Conclusion

Based on our results, patients with PID/SID or SOT/HSCT are susceptible to cancers. The types of incident cancer identified in our and prior studies suggest a limitation of routine single cancer screening tests that target only certain patient groups and address only five cancer types. A higher incidence of cancer has led to recommendations for more intensive cancer screening among SOT/HSCT patients in a few clinical guidelines [36]. Furthermore, earlier detection of cancer may be particularly valuable among patients with PID/SID and SOT/HSCT as cancer can be more challenging to treat among these patients due to the variety of cancer types that may develop and a higher risk of severe infections secondary to the chemotherapy regimens. Future studies in the form of disease and economic modeling may help delineate the clinical and economic value of implementing a broader cancer screening approach in elevated risk patient populations such as SOT/HSCT and PID/SID patients.

## Supplementary Information

**Additional file 1: Table S1.** Baseline characteristics of patients stratified by risk cohort. **Table S2.** Independent predictors of cancer in the overall cohort by Cox Regression Analysis. **Table S3.** Type of new primary cancer diagnosed in each cohort.

## Data Availability

Raw data are not publicly available due to restriction related to patient privacy or consent. Derived de-identified data can be available upon reasonable request from the corresponding author.

## Abbreviations

US: United States
USPTF: United States Preventive Service Task Force
MCED: Multi Cancer Early Detection
PID/SID: Primary Immunodeficiency Disorder/ Secondary Immunodeficiency Disorder
TNF-i: Tumor Necrosis Factor- inhibitor
AIDS: Acquired Immune Deficiency Syndrome
SOT/HSCT: Solid Organ Transplant/ Hematopoietic Stem Cell Transplant
EDW: Electronic Data Warehouse
HCI-TR: Huntsman Cancer Institute-Tumor Registry
SEER: Surveillance, Epidemiology and End Results
ICD-CM: International Classification of Disease-Clinical Modification
CCI: Charlson Comorbidity Index
BMI: Body Mass Index
AIC: Akaike Information Criteria
BIC: Bayesian Information Criteria
HTN: Hypertension
CVD: Cardiovascular Disease
ESRD: End Stage Renal Disease
DM: Diabetes Mellitus
COPD: Chronic Obstructive Pulmonary Disease
GI: Gastrointestinal
EBV: Epstein Barr Virus
HPV: Human Papilloma Virus
