# Supplementary material for: Cancer incidence in immunocompromised patients: a single-center cohort study
Source: BMC Cancer. 2023 Jan 9;23:33. doi: 10.1186/s12885-022-10497-4 (PMC9830873; doi:10.1186/s12885-022-10497-4)
Supplement: Supplementary file 1 — Additional file 1: Table S1. Baseline characteristics of patients stratified by risk cohort. Table S2. Independent predictors of cancer in the overall cohort by Cox Regression Analysis. Table S3. Type of new primary cancer diagnosed in each cohort. [file 12885_2022_10497_MOESM1_ESM.docx]

Table S1: Baseline characteristics of patients stratified by risk cohort

|  | SOT/HSCT | PID/SID | TNF-i | *P* value |
| --- | --- | --- | --- | --- |
|  | (N = 2982) | (N = 7542) | (N = 3363) |  |
| Gender, n (%) |  |  |  | <0.01 |
| Female | 1118 (37.5) | 3458 (45.8) | 2113 (62.8) |  |
| Male | 1864 (62.5) | 4084 (54.2) | 1250 (37.2) |  |
| Age in years, mean (SD) (at or close to index date) | 50.3 (14.4) | 50.4 (18.2) | 46.8 (15) | <0.01 |
| Age in years, n (%) |  |  |  | <0.01 |
| 65 and above | 513 (17.2) | 1938 (25.7) | 415 (12.3) |  |
| 50 to 64 | 1227 (41.1) | 1736 (23) | 1102 (32.8) |  |
| 18 to 49 | 1242 (41.6) | 3868 (51.3) | 1847 (54.9) |  |
| Race/ethnicity, n (%) |  |  |  | <0.01 |
| White | 2361 (79.2) | 5454 (72.3) | 2853 (84.8) |  |
| Hispanic/Latino | 315 (10.6) | 865 (11.5) | 248 (7.4) |  |
| African American | 47 (1.6) | 300 (4) | 22 (0.7) |  |
| American Indian/Alaskan Native | 63 (2.1) | 72 (1) | 53 (1.6) |  |
| Asian/Pacific Islander | 67 (2.2) | 183 (2.4) | 64 (1.9) |  |
| Unknown/Other | 129 (4.3) | 668 (8.9) | 123 (3.7) |  |
| BMI, mean (SD) (N=11,951) (at or close to index) | 28.2 (5.9) | 27.7 (7) | 29 (7.6) | <0.01 |
| BMI, n (%) |  |  |  | <0.01 |
| Less than 18.5 | 60 (2.01) | 224 (3.0) | 79 (2.4) |  |
| 18.5 to 24.9 | 709 (23.8) | 2306 (30.5) | 958 (28.5) |  |
| 25 to 29.9 | 833 (27.9) | 2070 (27.4) | 911 (27.1) |  |
| 30 and above | 846 (28.4) | 1835 (24.3) | 1120 (33.3) |  |
| Unknown | 534 (18.0) | 1107 (14.6) | 295 (8.8) |  |
| CCI score (in the 6 months pre-index), mean (SD) | 4 (2.4) | 1.7 (2.5) | 1 (1.4) | <0.01 |
| CCI score, n (%) |  |  |  | <0.01 |
| 6 and above | 682 (22.9) | 914 (12.1) | 68 (2.0) |  |
| 3 to 5 | 1256 (42.1) | 982 (13) | 273 (8.1) |  |
| 1 to 2 | 954 (32.0) | 1746 (23.2) | 1577 (46.9) |  |
| 0 | 90 (3.0) | 3900 (51.7) | 1446 (43.0) |  |
| Prior history of cancer | 1132 (38) | 570 (7.6) | 63 (1.9) | <0.01 |
| Reported comorbidities/risk factors that predispose to cancer, n (%) (in the 6 months pre-index) |  |  |  |  |
| GERD/ peptic ulcer | 786 (26.4) | 511 (6.8) | 153 (4.5) | <0.01 |
| Genetic susceptibility to cancer | 6 (0.2) | 5 (0.1) | 1 (0) | 0.06 |
| IBD | 81 (2.7) | 108 (1.4) | 349 (10.4) | <0.01 |
| Benign in-situ disease | 295 (9.8) | 430 (5.7) | 141 (4.2) | <0.01 |
| DM | 745 (25) | 669 (8.9) | 169 (5) | <0.01 |
| HTN/CVD/ESRD | 1910 (64.1) | 1299 (17.2) | 340 (10.1) | <0.01 |
| Pancreatitis | 7 (0.2) | 7 (0.1) | 1 (0) | 0.06 |
| COPD/asthma/pneumonia/  bronchitis | 397 (13.3) | 515 (6.8) | 172 (5.1) | <0.01 |
| Dermatitis/psoriasis | 22 (0.7) | 60 (0.8) | 423 (12.6) | <0.01 |
| Lupus/celiac disease | 61 (2) | 129 (1.7) | 54 (1.6) | 0.37 |
| Use of diuretics | 4 (0.1) | 45 (0.6) | 9 (0.3) | <0.01 |
| Use of oral contraceptives | 1 (0) | 0 (0) | 1 (0) | 0.21 |
| Renal cyst | 113 (3.8) | 75 (1) | 6 (0.2) | <0.01 |
| HPV/HBV/HCV infection | 117 (3.9) | 257 (3.4) | 32 (0.87) | <0.01 |
| Past cholecystectomy | 15 (0.5) | 4 (0.1) | 2 (0.1) | <0.01 |
| SD, Standard Deviation; BMI, Body Mass Index; CHF, Congestive Heart Failure; AIDS, Acquired Immune Deficiency Syndrome; HIV, Human Immunodeficiency Virus; MI, Myocardial Infarction; GERD, Gastroesophageal Reflux Disease; IBD, Inflammatory Bowel Disease; COPD, Chronic Obstructive Pulmonary Disease; HPV/HBV/HCV, Human Papilloma Virus/ Hepatitis B Virus/Hepatitis C Virus | | | | |

Table S2: Independent predictors of cancer in the overall cohort by Cox Regression Analysis

| Covariates | HR [95% CI] |
| --- | --- |
| Cohort |  |
| SOT/HSCT vs. TNF-i | 2.16 [1.63, 2.87] |
| PID/SID vs. TNF-i | 2.32 [1.8, 3.00] |
| Gender |  |
| Female vs. Male | 0.81 [0.7, 0.94] |
| Race/Ethnicity |  |
| Hispanic or Latino vs. White | 0.79 [0.61, 1.02] |
| AA/AS/PI/AN/AI vs. White | 1.01 [0.75, 1.36] |
| Unknown or other vs. White | 0.50 [0.32, 0.79] |
| Age |  |
| 50+ vs. 18 - 49 | 3.04 [2.57, 3.59] |
| BMI |  |
| <18.5 vs. 18.5 - 24.9 | 1.08 [0.67, 1.76] |
| 25 - 29.9 vs. 18.5 - 24.9 | 1.23 [1.01, 1.48] |
| ≥30 + vs. 18.5 - 24.9 | 1.10 [0.9, 1.34] |
| Cancer history vs. no cancer history | 1.38 [1.11, 1.71] |
| CCI score |  |
| 6 + vs. 1-2 | 1.52 [1.21, 1.91] |
| 3 - 5 vs. 1-2 | 1.40 [1.14, 1.72] |
| 0 vs. 1-2 | 0.74 [0.62, 0.90] |
| Comorbidities |  |
| GERD vs. no GERD | 1.15 [0.9, 1.47] |
| IBD vs. no IBD | 0.60 [0.36, 1.03] |
| Benign in-situ disease vs. no in-situ disease | 2.28 [1.82, 2.85] |
| DM vs. no DM | 1.40 [1.13, 1.73] |
| HTN/CVD/ESRD vs. no HTN/CVD/ESRD | 1.60 [1.37, 1.88] |
| Pancreatitis vs. no pancreatitis | 1.08 [0.15, 7.69] |
| COPD/asthma/pneumonia/bronchitis vs. no such disease | 1.55 [1.21, 1.99] |
| Dermatitis/psoriasis vs. no dermatitis/psoriasis | 0.61 [0.36, 1.03] |
| Lupus/Celiac disease vs. no disease | 1.33 [0.82, 2.14] |
| Use of diuretics vs. no use | 2.67 [1.27, 5.61] |
| Renal cyst vs. no cyst | 2.35 [1.51, 3.66] |
| HPV/HBV/HCV infection vs. no infection | 1.32 [0.91, 1.90] |
| Cholecystectomy vs. no cholecystectomy | 0.96 [0.14, 6.83] |
| AA, African American; AS, Asian; PI, Pacific Islander; AN, Alaskan Native; AI, American Indian; GERD, Gastroesophageal Reflux Disease; IBD, Inflammatory Bowel Disease; COPD, Chronic Obstructive Pulmonary Disease; HPV/HBV/HCV, Human Papilloma Virus/ Hepatitis B Virus/Hepatitis C Virus | |

Table S3: Type of new primary cancer diagnosed in each cohort

| Type of cancer | SOT/HSCT  N=161  n (%) | PID/SID  N=471  n (%) | TNF-i  N=69  n (%) |
| --- | --- | --- | --- |
| Bone | 0 | 3 (0.7) | 0 |
| Breast | 8 (5) | 36 (7.6) | 11 (15.9) |
| Connective/Soft tissue | 3 (1.9) | 3 (0.7) | 0 |
| Endocrine | 5 (3.1) | 8 (1.7) | 0 |
| GI | 23 (14.3) | 79 (16.8) | 8 (11.6) |
| Head | 14 (8.7) | 37 (7.9) | 10 (14.5) |
| Hematopoietic/ Reticulocyte system | 20 (12.4) | 105 (22.3) | 7 (10.1) |
| Oral | 19 (11.8) | 26 (5.5) | 5 (7.2) |
| Reproductive | 18 (11.2) | 56 (11.9) | 12 (17.4) |
| Respiratory | 13 (8.1) | 22 (4.7) | 1 (1.4) |
| Skin | 20 (12.4) | 67 (14.2) | 10 (14.5) |
| Urinary | 13 (8.1) | 20 (4.2) | 5 (7.2) |
| Ill-defined | 5 (3.1) | 9 (1.9) | 0 |
